# Highlight report: General determinants of steatosis

**DOI:** 10.17179/excli2018-2011

**Published:** 2018-12-19

**Authors:** Wiebke Albrecht

**Affiliations:** 1 IfADo - Leibniz Research Centre for Working Environment and Human Factors at TU Dortmund, Ardeystr. 67, D-44139 Dortmund, Germany

## ⁯⁯

Recently Christian Hudert and colleagues from the Charité in Berlin published a study about genetic determinants in pediatric non-alcoholic liver disease (Hudert et al., 2018[9]). Non-alcoholic fatty liver disease (NAFLD), the most frequent chronic liver disease in children, is known to be strongly influenced by genetic factors (Nobili et al., 2016[14]; Schwimmer et al., 2006[17]; Makkonen et al., 2009[12]; Anstee et al., 2016[2]). However, genetic determinants of a portal/zone-1 pattern of steatosis in children are not yet known. This would be important, because a portal/zone-1 pattern of steatosis leads to an increased risk of disease progression to fibrosis (Africa et al., 2018[1]; Mann et al., 2016[13]). To address this question, the authors established the Berlin adolescence NAFLD cohort (BaNA) and studied a set of single nucleotide polymorphisms. Interestingly, a variant of the retinyl-palmitate lipase PNPLA3 (rs738409) was associated with a periportal pattern of steatosis and also with an increased risk of progression to fibrosis (Hudert et al., 2018[9]). Therefore, obese children with the PNPLA3 variant may be candidates for a more intensive clinical follow-up and intervention.

Due to the current increase in the incidence of liver diseases a better understanding of their pathophysiology is of major importance (Jansen et al., 2017[11]; Vartak et al., 2016[20]; Hammad et al., 2014[7]; Hassan, 2016[8]; Stöber, 2016[18]; Bolt, 2017[3]; Ekhlasi et al., 2017[5]). For this purpose systems modeling as well as the analysis of expression patterns in relation to a phenotype represent frequently applied tools (Godoy et al., 2016[6]; Crespo Yanguas et al., 2016[4]; Jain et al., 2016[10]; Saleem et al., 2016[15]; Schenk et al., 2017[16]; Thiel et al., 2015[19]). The newly established BaNA cohort of adolescent NAFLD with its careful phenotyping and availability of proteome data is an important milestone for a better understanding of disease progression in steatosis. 
